# A Low-Profile and Ultra-Wideband Pancharatnam–Berry Coding Metasurface for High-Efficiency and Wide-Angle Circular Polarization Anomalous Reflection

**DOI:** 10.3390/ma17194730

**Published:** 2024-09-26

**Authors:** Cuizhen Sun, Junfei Gao, Huanhuan Gao, Xiongwei Ma, Xiaojun Huang

**Affiliations:** 1 College of Communication and Information Engineering, Xi’an University of Science and Technology, Xi’an 710054, China; scz@xust.edu.cn (C.S.); gjf1031@126.com (J.G.); mxw.980802@gmail.com (X.M.); 2 Engineering Research Center of Smart Coal Mine Advanced Communication Technology, Universities of Shaanxi Province, Xi’an 710054, China; 3 Air and Missile Defense College, Air Force Engineering University, Xi’an 710051, China; ghh1998kyjy@163.com

**Keywords:** polarizer, coding metasurface, circular polarization, anomalous reflection

## Abstract

The manipulation of electromagnetic waves using metasurfaces is important in areas such as stealth and communication. In this paper, we reported on the use of an element-based polarizer for the first step, which enables the incident electromagnetic waves to integrate into the cross-polarized waves with a relative bandwidth of 88% within 15–37.1 GHz. Then, an eight-element coding metasurface based on the Pancharatnam–Berry phase is presented for circular polarization anomalous reflection. The simulated values show that our work can achieve a high-efficiency (94%) and wide-angle (70°) anomalous reflection under normal incidence. The simulated values present good agreement with the experimental values. Our work reveals the ability to manipulate the waves and electromagnetic stealth.

## 1. Introduction

The manipulation of electromagnetic (EM) waves in desired ways hold significant prospects in various fields such as communication and stealth [1,2]. Metasurfaces, composed of sub-wavelength artificial elements, have the capability to reshape the wavefront of EM waves by imparting them with arbitrary phase distributions [3,4]. This provides an effective method for wavefront manipulation and enables a variety of applications, including in holograms [5], metalens [6], and vector beams [7]. As one of the simplest phenomena in wave manipulation, anomalous reflection reflects the incident electromagnetic wave to a desired direction [8]. However, due to the impedance mismatch between the incident wavefront and the desired wavefront, implementing abnormal reflection based on the generalized formula, Snell’s law (local response), often results in parasitic reflections in non-desired directions, making it difficult to achieve high-efficiency and wide-angle anomalous reflection simultaneously [9].

Recently, researchers have been devoted to improving the efficiency and wide-angle performance of anomalous reflections of linearly polarized waves [10,11,12]. For example, metamaterial-inspired diffraction gratings have attracted lots of attentions due to the unprecedented high efficiency in anomalous reflection and arbitrary beam splitting by means of relatively simple configurations [12]. Meanwhile, some related theories also have been proposed to improve performance [13]. Furthermore, the control of circularly polarized waves has a wide range of applications in improving communication quality, enhancing radar and wireless navigation, and so on [14,15]. Therefore, improving the anomalous reflection performance of circularly polarized waves is also a key issue to be addressed. However, it should be noted that the specific aspects of reflection efficiency and angle performance may not have been involved in the referenced paper. Instead, the focus may have been more on bandwidth improvement [16,17]. The Pancharatnam–Berry (PB) phase metasurface has attracted significant attention due to its effective manipulation of circularly polarized waves [18,19]. By simply rotating the metasurface, any desired phase variation can be introduced in cross-polarization, greatly simplifying the design process for improving efficiency and bandwidth. In 2019, anomalous reflection was realized for two circular polarizations independently in the 12–18 GHz frequency range [20]. In 2020, two PB unit cells were designed and manufactured to achieve the spin Hall effect and focusing effect within the frequency range of 8.2 to 17.3 GHz [21]. In 2023, a double-arrow-shaped resonator structure was proposed to achieve beam deflection within 0.45–1.75 THz [22]. Therefore, achieving a balance between bandwidth, efficiency, and angle performance for anomalous reflections of circularly polarized waves is a key area of focus and challenge.

In this paper, a low-profile reflective metasurface is designed to achieve polarization conversion [23,24], which enables the incident electromagnetic waves into the cross-polarized waves with the relative bandwidth of 88% within 15–37.1 GHz. Moreover, coding metasurfaces based on the PB phase principle were optimized for achieving high-efficiency (94%) and wide-angle (70°) anomalous reflection. The experimental results are consistent with the simulated results, which also shows the effectiveness of the arithmetic for optimizing the coding metasurface to achieve anomalous reflection. This study also offers a practical solution for beam manipulation, stealth, and other applications.

## 2. Method and Design

We define *u*-polarization as a plan in the *x* direction, and *v*-polarization is in the y direction perpendicular to it. Based on the reflection theory, cross-polarization does not exist in *u*- and *v*-polarization incidence due to the symmetry of the anisotropic unit cell [8]. When *u*- and *v*-axes overlap with the *x*- and *y*-axes, *r_xx_* = *r_uu_*, *r_yy_* = *r_vv_*. The angle of rotation between the positive *u*-axis and *x*-axis is defined as *φ*, and the reflection matrix can be described as

(1) $$R_{lin}={\left(\begin{array}{cc} \cos\varphi & -\sin\varphi \\ \sin\varphi & \cos\varphi \end{array}\right)}^{-1}\left(\begin{array}{cc} r_{uu} & 0\\ 0 & r_{vv} \end{array}\right)\left(\begin{array}{cc} \cos\varphi & -\sin\varphi \\ \sin\varphi & \cos\varphi \end{array}\right)$$

At this point, the circularly polarized incident reflection matrix is

(2) $$R_{cir}=\frac{1}{2}\left(\begin{array}{l} 1\;\;\;-j\\ 1\;\;\;\;j \end{array}\right)R_{lin}{\left(\begin{array}{l} 1\;\;\;-j\\ 1\;\;\;\;j \end{array}\right)}^{-1}=\left(\begin{array}{l} r_{-+}\;\;r_{--}\\ r_{++}\;\;r_{+-} \end{array}\right)$$

where the right- and left- handed states of circularly polarized waves are denoted by subscripts + and −, respectively. The gradient metasurface can be designed in conjunction with the PB phase theory and play a part in the anomalous reflection of circularly polarized waves. The reflection amplitude of the circularly polarized waves remains unchanged and realizes a reflection phase shift of ±2*φ*; therefore, the following phase gradient would be produced:

(3) $$\nabla \alpha =\frac{\pm 2\varphi }{P}=\frac{\pm 2\pi }{L}$$

where *L* = N*P*, N represents the amount of subunit structures, and *P* represents the phase gradient. When the incident wave is vertically incident ($\theta_{i}=0$), the anomalous reflection angle is expressed as follows [18]:

(4) $$\theta_{r}=\frac{k_{0}\sin\theta_{i}+\nabla \alpha }{k_{0}}$$

Here, $k_{0}=2\pi /\lambda$ represents the wave vector in free space, and $\theta_{i}$ denotes the angle of incidence. t can be simplified a $\theta_{r}=\arcsin(\pm \lambda /L)$.

Then, a coding metasurface consisting of M × M array elements is introduced, where each array element consists of the M × M array of basic units. Due to the destructive elimination of the coding metasurface, the plane wave is positively incident, and the far-field function can be indicated as

(5) $$F(\theta ,\varphi )=\sum_{m=1}^{N}\sum_{n=1}^{N}\exp\left\{-i\left[\begin{array}{l} \varphi_{m,n}+k_{0}D(m-1/2)\sin\theta \cos\varphi \\ +k_{0}D(n-1/2)\sin\theta \sin\varphi \end{array}\right]\right\}$$

## 3. Design and Simulation

**Figure 1:**
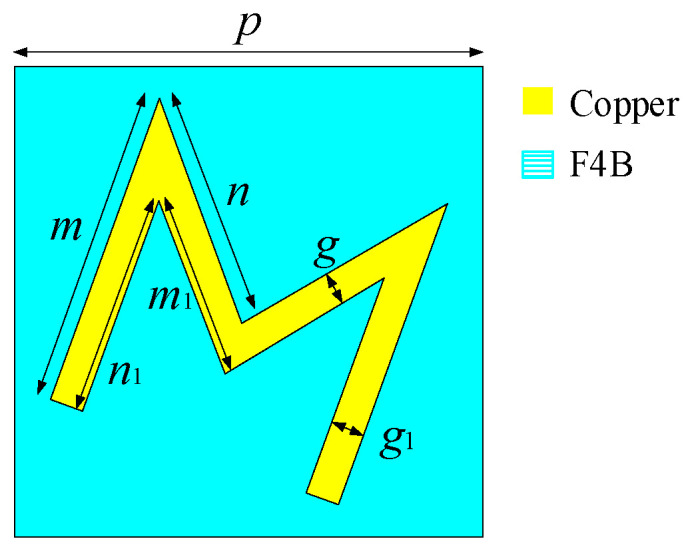
The proposed circular polarizer.

The designed low-profile and ultra-wideband circular polarizer is shown in Figure 1, which consisted of a metal pattern layer, a metal backplate, and a dielectric layer. The M-shaped metal pattern and backplate are both made of copper films with a thickness of 0.035 mm and a conductivity of 5.8 × 10^7^ S/m. The dielectric layer is composed of F4B material, characterized by a dielectric constant of 3 and a tangent loss of 0.025; the F4B sheet is a polytetrafluoroethylene (PTFE)-based composite material with an extremely low dielectric constant and loss tangent value, and the F4B sheet also has good chemical stability and temperature stability. The optimized dimensions of the polarizer are *p* = 4 mm, m = 2.64 mm, n = 2.52 mm, m_1_ = 1.5 mm, n_1_ = 1.8 mm, g = 0.36 mm, g_1_ = 0.48 mm, l = 2.1 mm, and l_1_ = 1.5 mm.

**Figure 2:**
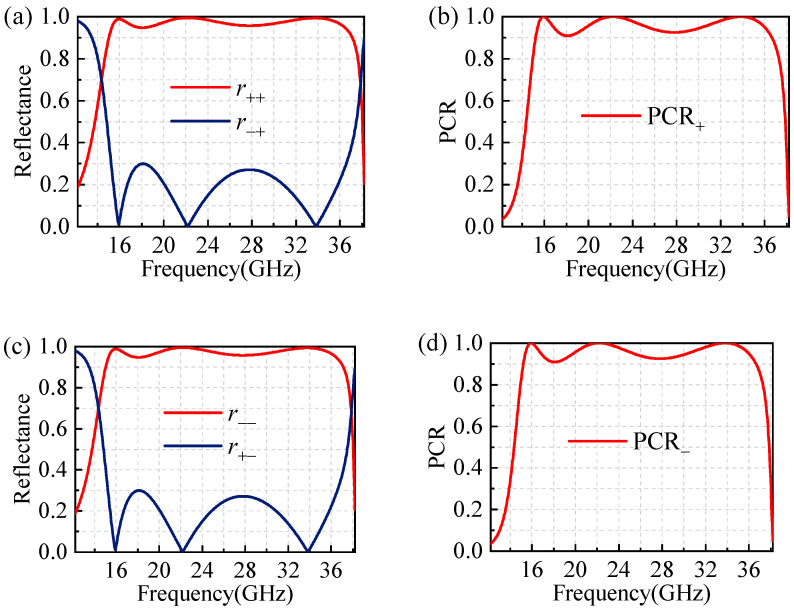
The reflection coefficient and PCR under the right- and left-handed circularly polarized wave. (**a**) *r*_++_ and *r*_−+_. (**b**) PCR_+_. (**c**) *r*_−−_ and *r*_+−_. (**d**) PCR_−_.

We use the full-wave simulation software CST Studio Suite v2022 to investigate the capability of this polarizer to convert incident circularly polarized waves into cross-polarized waves. The boundary conditions are set as unit cell in the *x* and *y* directions, while an open boundary is assigned in the +*z* direction. The reflection coefficients of simulated cross-polarized and co-polarized waves are shown in Figure 2. When a right-handed circularly polarized wave is normally incident, it will be cross-polarized in the form of right-handed circularly polarized waves through the polarizer. The reflectance of *r*_++_ and *r*_−+_ in 12.2–38.2 GHz are depicted in Figure 2a. As can be seen, the reflection coefficient *r*_++_ is greater than 0.9 in 15–37.1 GHz, while the reflection coefficient *r*_−+_ is less than 0.9 in 15–37.1 GHz. Meanwhile, the reflectance of *r*_++_ and *r*_−+_ exist at three resonant points: 15.8 GHz, 22.2 GHz, and 33.9 GHz, respectively. Then, the polarization conversion rate (PCR) is calculated by PCR = |*r*_++_|^2^/(|*r*_++_|^2^+|*r*_+−_|^2^). It is observed that PCR is above 0.9 in 15–37.1 GHz with a relative bandwidth of 88% in Figure 2b. In particular, the PCR at the three resonant points is almost 1, which signifies a generation of perfect polarization conversion. Likewise, when a left-handed circularly polarized wave is normally incident, the reflection coefficient and PCR of the polarizer are shown in Figure 2c,d. It is also obvious that polarization conversion occurs through the designed polarizer within an ultra-wideband range.

**Figure 3:**
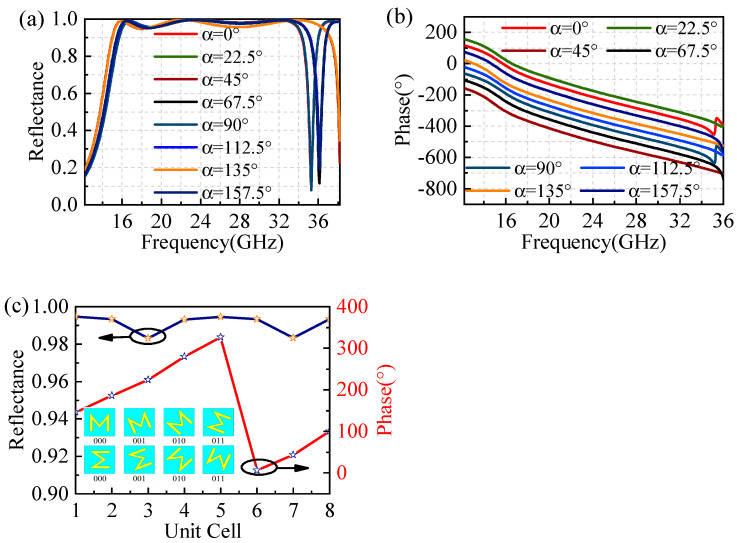
The reflectance and phase. (**a**) Reflectance and (**b**) phase in 15–34 GHz. (**c**) Reflectance and phase at 24 GHz.

In order to investigate the circular polarization anomalous reflection utilizing this low-profile and ultra-wideband polarizer, according to the PB phase theory, it is evident that there is a phase difference of 45° between adjacent units by rotating the polarizer with a step size of 22.5°. The reflection coefficients and phase of these eight units are displayed in Figure 3. When right-handed circularly polarized waves are normally incident, the reflectance of these eight units remains almost consistent in Figure 3a, except for some differences near the frequency of 36 GHz. Obviously, the phase achieves full coverage from 0° to 360° in Figure 3b. Therefore, these eight units within the range of 15–34 GHz can be encoded to achieve the circular polarization anomalous reflection. As shown in Figure 3c, at 24 GHz, these eight units are encoded as “000”, “001”, “010”, “011”, “100”, “101”, “110”, and “111”, respectively. The corresponding phase values for these encoded units are 272.2°, 181.2°, 97.5°, 0.75°, 272.2°, 181.2°, 97.5°, and 0.75°, with a phase difference of approximately 45°. At the same time, all the reflection coefficients are greater than 0.98 to achieve high-efficiency anomalous reflection.

## 4. Optimization of Coding Metasurface

**Figure 4:**
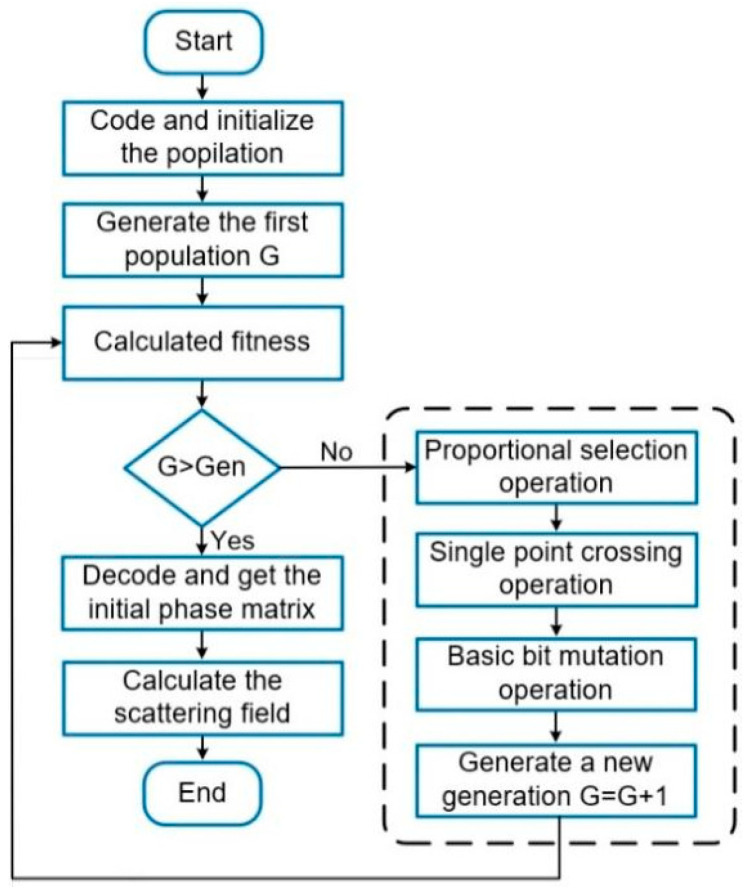
The flowchart of the GA optimization.

GA is a global optimization algorithm that simulates the process of biological evolution. This work employs the GA to optimize the layout of the 2-bit coding metasurface for achieving arbitrary and efficient anomalous reflection. The optimization process of the GA can be broadly categorized into four key stages: encoding, selection, crossover, and mutation. Figure 4 shows the flowchart of GA optimization. It can be summarized into the following steps: (1) encode and generate an initial population based on the input phase; (2) calculate the fitness of each individual and select the fittest individuals from the population; (3) determine whether the convergence criteria are met; (4) if the convergence criteria are met, decode and terminate the optimization process; otherwise, perform GA operations (selection, crossover, and mutation), and then return to steps (2)–(4).

**Figure 5:**
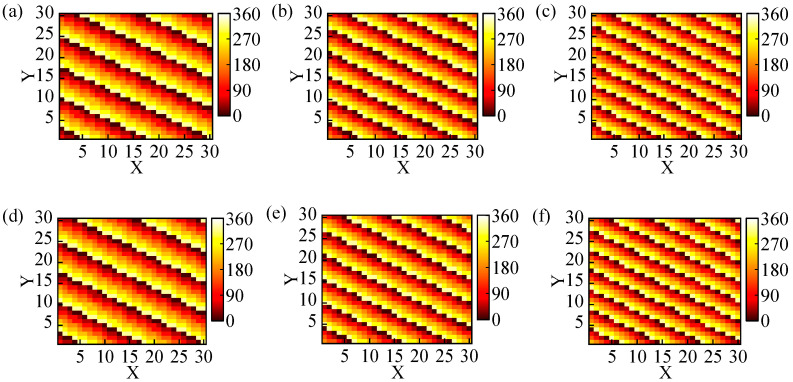
The phase and 3D far-field distribution of three anomalous reflection coding metasurfaces: (**a**,**d**) (30°, 330°), (**b**,**e**) (40°, 270°), and (**c**,**f**) (50°, 330°).

**Figure 6:**
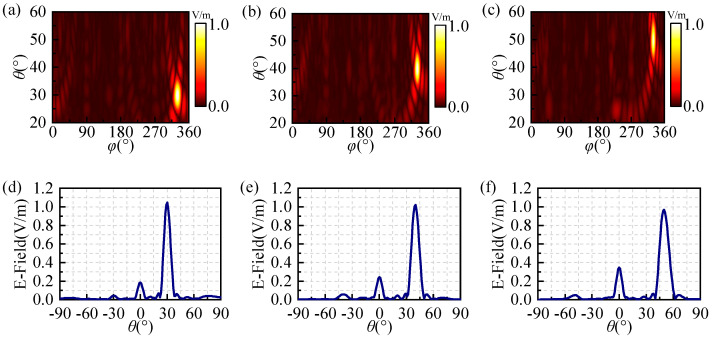
The 2D far-field distribution and E-field density of three anomalous reflection coding metasurfaces: (**a**,**d**) (30°, 330°), (**b**,**e**) (40°, 330°), and (**c**,**f**) (50°, 330°).

Here, we initially employed genetic algorithms to optimize the coding metasurfaces, enabling the realization of anomalous reflections of circularly polarized beams with θ = 30°, 40°, 50°, and φ = 330°. Considering the computational cost implications, our study focused on investigating a 30 × 30 array of coding metasurfaces. The phase distribution of the optimized coding metasurfaces and simulated 3D far-field patterns at 24 GHz are illustrated in Figure 5. It can be clearly observed that the incident right-handed circularly polarized plane wave is reflected in a specific direction in Figure 6d–f, indicating that the optimized coding metasurfaces have achieved the anomalous reflection of circularly polarized waves. Meanwhile, the angles corresponding to the anomalous reflection beams can be observed in Figure 6a–c. In this 2D far-field, the brightest regions correspond to the beams exhibiting anomalous reflection and the angles of the three beams are *θ* = 30°, 40°, 50°, and *φ* = 330°. The E-field densities of these three beams are presented in Figure 6d–f. The three optimized coding metasurfaces exhibit a strong electric field at the angles of anomalous reflection. There are several peaks in Figure 6 that are generated by harmonics that are negligible relative to the main peaks, which are caused by the coupling between the metasurface elements themselves, and this specular reflection is unavoidable. In order to evaluate the efficiency of beam anomalous reflection, we calculate the ratio between the energy of anomalous reflection and the total scattered energy in space based on the obtained results. The efficiency of anomalous reflection in the three directions *θ* = 30°, 40°, and 50° is calculated to be 94%, 93%, and 92%, respectively. Therefore, it can achieve high-efficiency anomalous reflection within the ultra-wideband range by optimizing the coding metasurfaces with GA.

**Figure 7:**
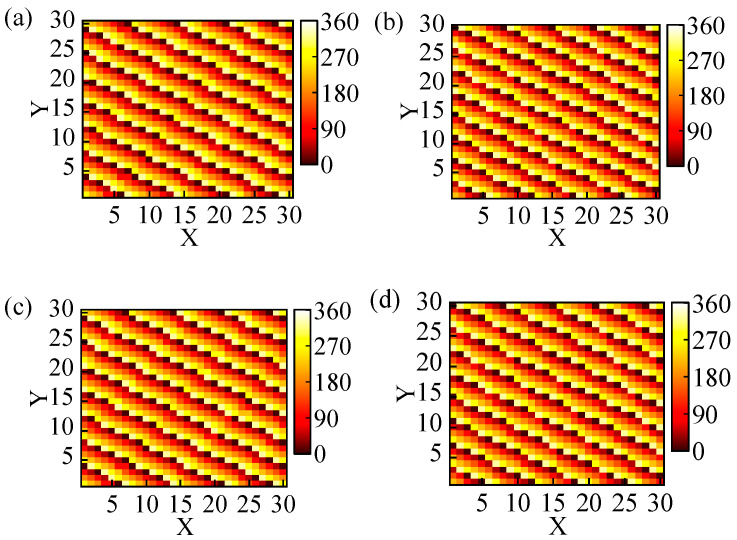
The two anomalous reflections encode the surface layout and far-field distribution of the element: (**a**,**c**) (60°, 330°) and (**b**,**d**) (70°, 330°).

**Figure 8:**
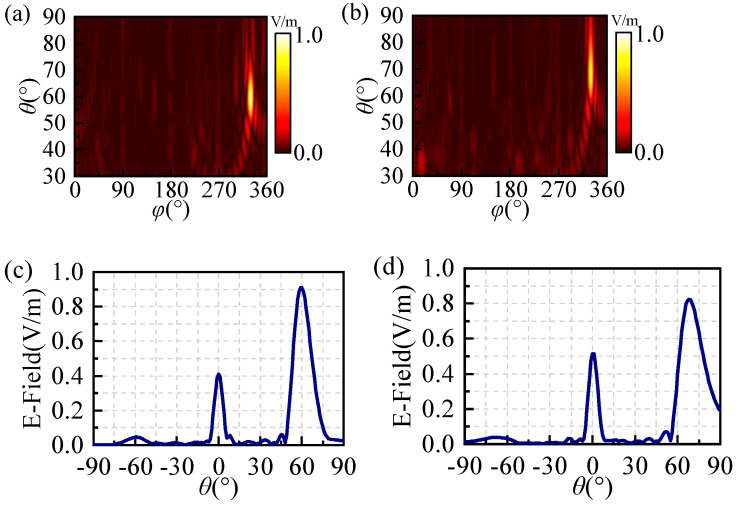
Two anomalous reflections encode the E-field density and two-dimensional far-field distribution on the surface of the element: (**a**,**c**) (60°, 330°) and (**b**,**d**) (70°, 330°).

In order to investigate the circularly polarized anomalous beam reflection with wide angles and high efficiency achieved by utilizing coding metasurfaces optimized with GA, we utilize two 30 × 30 coding metasurface arrays to achieve anomalous beam reflection with *θ* = 60°, 70°, and *φ* = 330°. The phase distribution of the optimized coding metasurfaces and simulated 3D far-field patterns at 24 GHz are illustrated in Figure 7. We can clearly observe that the incident right-handed circularly polarized plane wave is reflected in a specific direction in Figure 8c,d, despite the increased specular reflection of the beam with *θ* = 70°; this can also serve as evidence of the successful achievement of circular polarization anomalous reflection. Meanwhile, the angles corresponding to the anomalous reflection beams can be obtained in Figure 8a,b. In this 2D far-field, the brightest regions correspond to the beams exhibiting anomalous reflection and the angles of the three beams are *θ* = 60°, 70°, and *φ* = 330°. The E-field densities of these two beams are shown in Figure 8c,d. Similarly, the two optimized coding metasurfaces generate a strong electric field at the angles of anomalous reflection. The efficiency of anomalous reflection in the two directions *θ* = 60° and 70° is calculated to be 89% and 70%, respectively. Therefore, it can achieve wide-angle and high-efficiency anomalous reflection within the ultra-wideband range by optimizing the coding metasurfaces with GA.

## 5. Experimental Verification

**Figure 9:**
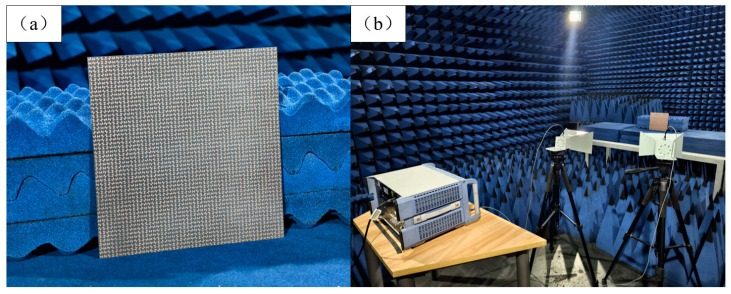
(**a**) The coding metasurface sample. (**b**) Experimental environment.

To validate the effective realization of anomalous reflections of circularly polarized waves using genetic-algorithm-optimized coding metasurfaces, a coding metasurface sample with 50 × 50 units and a size of 20 × 20 cm^2^ is fabricated and measured, which is shown in Figure 9a. Subsequently, the reflection coefficients in each direction were measured using a turntable. The experimental platform is shown in Figure 9b.

**Figure 10:**
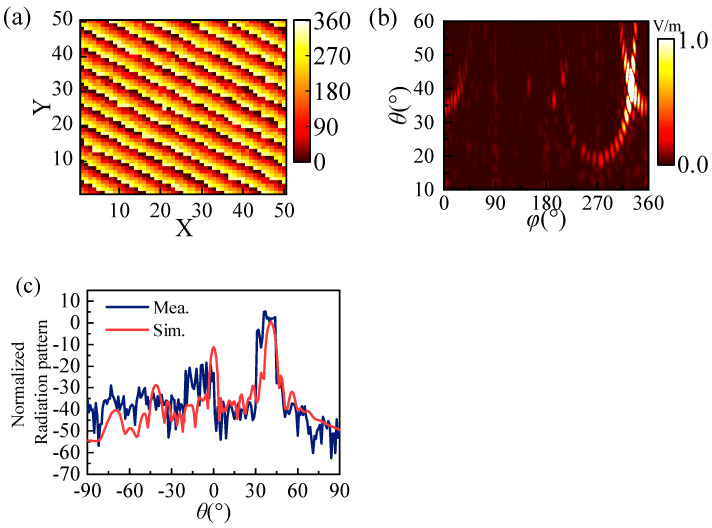
Simulated and experimental results. (**a**) The layout distribution of the sample. (**b**) The simulated far-field result. (**c**) Radiation pattern of simulated and experimental results.

The coding metasurface sample is optimized to achieve the desired beam direction with *θ* = 40° and *φ* = 330°. The simulated and experimental results are presented in Figure 10. Figure 10a,b display the layout of the coding metasurface as well as its corresponding far-field distribution, allowing for a clear observation that the incident plane is reflected in a specific direction. Figure 10c shows the comparison between the simulated results of the coding metasurface and the experimental results. It is evident that there is good agreement between the results.

## 6. Conclusions

In conclusion, a new low-profile and ultra-wideband reflection polarizer has been proposed and measured for applying high-efficiency (94%) and wide-angle (70°) anomalous reflection under normal incidence. The basic unit enabled the linear polarization wave to convert into a cross-polarized state with a relative bandwidth of 88% and a PCR of almost 90 from 15 GHz to 37.1 GHz. Coding metasurfaces based on the PB phase principle were designed to react from circular polarization anomalous reflection. The simulated values have good agreement with the experimental values. Our work reveals the ability to manipulate the waves and electromagnetic stealth. This indicates that the ability to manipulate polarized waves plays an important role in electromagnetic stealth and antenna designs.

## Data Availability

The original contributions presented in the study are included in the article, further inquiries can be directed to the corresponding author.
